# Phosphorylation of the androgen receptor is associated with reduced survival in hormone-refractory prostate cancer patients

**DOI:** 10.1038/sj.bjc.6604152

**Published:** 2008-03-18

**Authors:** P McCall, L K Gemmell, R Mukherjee, J M S Bartlett, J Edwards

**Affiliations:** 1 Section of Surgery, Division of Cancer Sciences and Molecular Pathology, Glasgow Royal Infirmary, Glasgow, UK

**Keywords:** Akt, androgen receptor, hormone-refractory prostate cancer

## Abstract

Cell line studies demonstrate that the PI3K/Akt pathway is upregulated in hormone-refractory prostate cancer (HRPC) and can result in phosphorylation of the androgen receptor (AR). The current study therefore aims to establish if this has relevance to the development of clinical HRPC. Immunohistochemistry was employed to investigate the expression and phosphorylation status of Akt and AR in matched hormone-sensitive and -refractory prostate cancer tumours from 68 patients. In the hormone-refractory tissue, only phosphorylated AR (pAR) was associated with shorter time to death from relapse (*P*=0.003). However, when an increase in expression in the transition from hormone-sensitive to -refractory prostate cancer was investigated, an increase in expression of PI3K was associated with decreased time to biochemical relapse (*P*=0.014), and an increase in expression of pAkt^473^ and pAR^210^ were associated with decreased disease-specific survival (*P*=0.0019 and 0.0015, respectively). Protein expression of pAkt^473^ and pAR^210^ also strongly correlated (*P*<0.001, c.c.=0.711) in the hormone-refractory prostate tumours. These results provide evidence using clinical specimens, that upregulation of the PI3K/Akt pathway is associated with phosphorylation of the AR during development of HRPC, suggesting that this pathway could be a potential therapeutic target.

Prostate cancer is an increasing heath-care problem, one out of six UK men are diagnosed with this disease, which ranks second after lung cancer as a cause of male cancer-specific mortality (Cancer Research UK, 2004). Advanced prostate cancer treatment has relied on hormone-deprivation therapy for the past 50 years. Response rates are initially high (70–80%); however, almost all patients relapse and develop hormone-refractory prostate cancer (HRPC), resulting in increased morbidity and death (Cleeve and Goad, 1995).

Amplification of the androgen receptor (AR) may explain development of HRPC in 20–30% of patients (Edwards *et al*, 2001). *In vitro* studies demonstrate that Akt/protein kinase B phosphorylates AR at serine residues (Ser^210^ and Ser^790^) resulting in modulation of AR transcriptional activity (Lin *et al*, 2001, 2003) and suggesting that AR phosphorylation might promote development of HRPC. It has also been reported that activation of phosphatidylinositol 3-OH kinase (PI3K)/Akt pathway can induce expression of AR at the protein and mRNA level, again suggesting that this pathway may be involved with hormone-refractory disease (Yang *et al*, 2005). Akt is a downstream member of the PI3K cascade, which plays an important role in cell growth, death, adhesion and migration and is frequently activated in cancer cells (Lin *et al*, 2003). Akt is activated when phosphorylated at threonine 308 and subsequently serine 473. There are three members of the Akt family, Akt1, -2 and -3, and evidence suggests that the three isoforms of Akt have different roles in the development of hormone-resistant breast cancer either via interactions with the oestrogen receptor or via proteins involved in proliferation and apoptosis, for example, mTOR and BAD (Kirkegaard *et al*, 2005). Akt may play a similar role in the development of HRPC (Liao *et al*, 2003). Cell line studies demonstrate that low passage LNCaP cells (hormone-sensitive prostate cancer cells) have low basal Akt activity, possibly due to early-stage prostate cancer relying on hormones for growth and survival. However, during hormone ablation or antihormone treatment, LNCaP cells undergo growth arrest and apoptosis, and Akt activity is upregulated (more than 20-fold higher), resulting in stimulation of cell growth, compensating for the effects of androgen withdrawal (Gao *et al*, 2003). Data from these experiments suggest that Akt signals for cell growth and survival at low levels of androgen, and therefore may promote development of HRPC (Ghosh *et al*, 2003; Lin *et al*, 2003). This is supported by a report that demonstrates that upregulation of the PI3K cascade allows cells to grow in the presence of antiandrogens and contributes to failure of endocrine therapy (Murillo *et al*, 2001).

A recent review suggests that the PI3K/Akt pathway is a possible therapeutic target for treatment of prostate cancer (Pommery and Henichart, 2005). Prostate tumours are reported to have significantly higher Akt expression than BPH (Liao *et al*, 2003), and only 10% of well-differentiated prostate tumours strongly express pAkt compared to 92% of poorly differentiated tumours (Malik *et al*, 2002; Ayala *et al*, 2003; Ghosh *et al*, 2003). Akt1 and Akt2 expression in hormone-sensitive tumours have been associated with shorter time to biochemical relapse; however, no association was reported with the activated forms or with survival (Le Page *et al*, 2006). However to our knowledge, this is the first study to conduct a comprehensive investigation into the changes that occur to multiple members of the pathway in the transition from clinical hormone-sensitive to -refractory prostate cancer, and in particular, to test if phosphorylation of AR is associated with patient outcome measures.

## MATERIALS AND METHODS

### Patient

Sixty-eight patients with matched hormone-sensitive and -refractory tumour pairs were retrospectively selected for analysis. All tumours had patient identification removed, including block number and hospital number and were coded to make the database anonymous. Ethical approval was obtained from the Multicentre Research Ethics Committee for Scotland (MREC/01/0/36) and Local Research and Ethical Committees. Patients were only selected for analysis if they initially responded to hormone treatment (in the form of subcapsular bilateral orchidectomy or maximum androgen blockade), but subsequently relapsed (two consecutive rises in PSA greater than 10%) and had a pre- and posthormone relapse tissue sample available for analysis.

### Immunohistochemistry

All IHC was performed on 5 *μ*m, archival formalin-fixed, paraffin-embedded prostate tumour sections on separate slides. Immunohistochemistry for PI3K (p110 catalytic subunit), Akt1–3, phosphorylated Akt at threonine 473 (pAkt^473^), mTOR, phosphorylated mTOR at serine 2448 (mTOR^2448^) and phosphorylated AR at serine 210 (pAR^210^) were performed as follows: antigen retrieval was performed using heat treatment under pressure in a Tris EDTA buffer (5 mM Trizma Base, 1 mM EDTA, pH 8: Akt1–3 and pAR^210^) or citrate buffer (PI3K and mTOR) for 5 min or by heating to 96°C for 20 min in citrate buffer (pAkt^308^, pAkt^308^ and pmTOR^2448^). Nonspecific background staining was blocked using either 2.5% horse serum in TBS for 20 min (PI3K and pAR^210^), in 1% casein for 10 min (Akt1–3, mTOR and pmTOR^2448^) or in Serum Free Block (Dako A/S, Glostrup, Denmark) for 10 min (pAkt^473^). Phosphatidylinositol 3-OH kinase (Cell Signalling Technology, Beverly, MA, USA), Akt1–3 (Santa Cruz Biotechnology Inc., Santa Cruz, CA, USA), pAkt^473^ (Cell Signalling Technology), mTOR (Santa Cruz Biotechnology Inc.), pmTOR^2448^ (Cell Signalling Technology) and pAR^210^ (Imgenex, San Diego, CA, USA) antibodies were used at the following concentrations (1, 1, 2, 2, 4, 5, 2 and 50 *μ*g ml^−1^). Phosphorylated AR at serine 210 was incubated for 1 h at 25°C and all other antibodies were incubated overnight at 4°C. For the mTOR antibody only, incubation with rabbit anti-goat antibody (Dako A/S) (1 : 4000) for 1 h at room temperature was also required. Staining for PI3K, pAkt^308^, pAkt^473^, mTOR and pmTOR^2448^ was developed using EnVision plus kit (Dako A/S) and staining for Akt1–3 and pAR^210^ was developed using LSAB kit (Dako A/S) and 3,3-diaminobenzidine tetrahydrochloride (DAB; Vector Laboratories, CA, USA). Nuclei were counterstained with haematoxylin before mounting. A positive and a negative control were included in each IHC run, negative controls were incubated with an isotype-matched control antibody at a concentration of 1 mg ml^−1^. Positive control slides were breast tissue known to express PI3K, Akt and mTor, BPH tissue known to express AR and MCF-7 and LNCaP cell pellets. Antibody specificity was confirmed by western blotting. In addition, phosphorylated antibodies were confirmed to detect only the phosphorylated forms using calf intestinal alkaline phosphatase to destroy phosphorylated proteins. Two identical slides had IHC performed on them, the only difference being that one was previously treated with calf intestinal alkaline phosphatase, the untreated slide expressed the phosphorylate protein and the treated slide did not, this technique confirms that the antibody is only detecting the phosphorylated form of the protein and not the unphosphorylated form.

Tissue staining intensity was scored blind by two independent observers using a weighted histoscore method (Fraser *et al*, 2003) also known as the Hscore system (McCarty *et al*, 1986). Histoscores were calculated from the sum of (1 × % cells staining weakly positive)+(2 × % cell staining moderately positive)+(3 × % cells staining strongly positive) with a maximum of 300. The interclass correlation coefficient (ICCC) for each protein was calculated to confirm consistency between observers, and the mean of the two observers' scores were used for analysis. Changes in staining between pre- and posthormone-refractory cases were defined as an increase or decrease with the 95% confidence interval (CI) for the difference in interobserver variation, which is the mean difference between the histoscores that each observer assigns for protein expression plus 2 s.d. Change in expression of PI3K, Akt1–3, mTOR, pmTOR^2448^ and pAR^210^ are shown in Table 1.

### Statistical analysis

Interclass correlation coefficients were used to confirm consistency between observers. Protein expression data were not normally distributed and are shown as median and interquartile ranges. Wilcoxon signed-rank tests were used to compare expression between pre- and posthormone-refractory tumours. Survival analyses were conducted using Kaplan–Meier method, and curves were compared with the log-rank test. Hazard ratios (HRs) were calculated using Cox regression analysis. Correlations between members of the pathway were performed using a Spearman's rank test.

## RESULTS

### Patients

A total of 68 prostate cancer patients (diagnosed between 1984 and 2000) were included in this study with matched hormone-sensitive (hormone-sensitive tissue was obtained from 26 patients by TRUS-guided biopsy and the remaining 42 by TURP) and -refractory prostate tumours (all obtained by TURP) available for analysis (136 tumours in total). Patients in this cohort were diagnosed with locally advanced (50) or metastatic prostate cancer (18) and subsequently received surgery and androgen deprivation therapy (26 subcapsular bilateral orchidectomy, 44 GnRH analogue and 2 had both). Forty-five of the sixty-eight patients also received antiandrogen therapy, and this included all those who received GnRH analogues. At initial diagnosis, the median age was 70 (66–74) years and 26% of patients had metastatic disease. The median time to biochemical relapse was 2.32 (1.48–4.00) years, and the percentage of patients with metastatic disease had increased to 57%. Sixty-one patients (89.7%) died during follow-up and median survival for these patients was 4.34 (2.94–6.63) years. Seven patients were alive at last follow-up; the median time of follow-up for all 68 patients was 4.34 (2.86–6.74) years.

### Protein expression patterns

**Figure 1:**
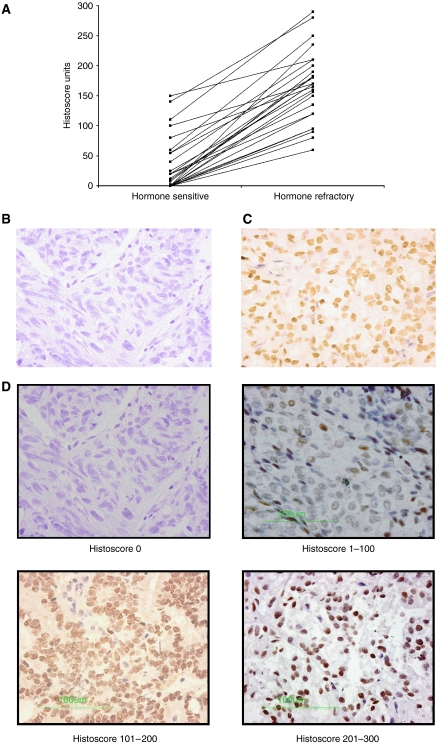
(**A**) shows the actual increase in histoscore units of the 42% of patients whose tumours exhibited an increase in pAR^210^ expression in the transition from hormone-sensitive to -refractory disease. (**B** and **C**) These images are of an actual pair of hormone-sensitive and -refractory tumours whose expression increased by 150 histoscore units. Brown nuclear staining denotes pAR^210^ expression. Magnification × 400. (**D**) These images show examples of a negative control, a tumour that was score with a histoscore in the range of 1–100, 101–200 or 201–300.

Akt1, pAkt^473^ and pmTOR^2448^ protein expression was observed in the cell membrane, cytoplasm or nucleus. mTOR expression was observed at the membrane and cytoplasm, and PI3K, Akt2 and Akt3 expression was observed only in the cytoplasm. Nuclear expression was observed for pAR^210^ (Figure 1; Table 1). To assess the level of agreement between observers, ICCCs were calculated for each antibody at each location using SPSS; all ICCC values in this study were >0.7 (which is classed as excellent) (Table 1).

#### Protein expression levels and changes in protein expression

The median expression levels for all proteins investigated were calculated for hormone-sensitive and -refractory tumours (Table 1). The Wilcoxon signed-rank test was used to compare expression levels in the hormone-sensitive tumours compared to hormone-refractory tumours. Using this method, only pAR^210^ significantly increased with the development of hormone-refractory disease. The median expression of pAR^210^ in hormone-sensitive tumours was 35 (0–85) increasing to 103 (50–169) in hormone-refractory tumours (*P*<0.0001) (Table 1). This demonstrates an increase in AR phosphorylation at the Akt consensus site in the transition from hormone-sensitive to -refractory disease.

The nature of our cohort, however (matched hormone-sensitive and -refractory tumours for each patient), allowed us to establish if there was a change in protein expression levels in the transition from hormone-sensitive to -refractory disease for each individual patient. By examining the change in protein expression for each patient, we were able to create subgroups of patients whose tumours exhibited either a fall or rise in protein expression for all proteins investigated (Table 1). Using this technique, it was observed that 42% of patients investigated in this study had an increase in pAR^210^ expression in the transition from hormone-sensitive to -refractory disease (Figure 1).

#### Are protein expression levels in hormone-sensitive or -refractory tumours associated with relapse or survival?

When expression levels of each protein investigated in the hormone-sensitive tumours were divided into high or low expression (levels above or below the median), none of the proteins investigated were associated with time to relapse or disease-specific survival. The histoscores used as a cutoff for each analysis was the median histoscore; the median histoscore for each protein investigated is given in Table 1.

**Figure 2:**
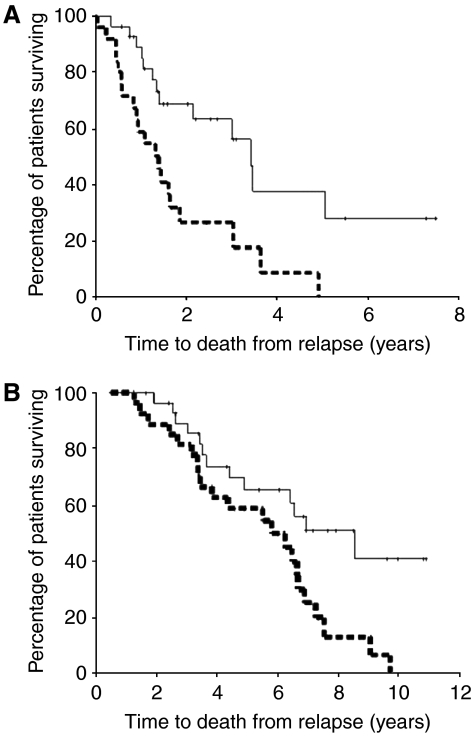
(**A**) Kaplan–Meier plot demonstrates that those patients whose hormone-refractory tumour has high pAR^210^ nuclear expression (broken line) have shorter time to disease-specific death from time of biochemical relapse than those patients whose hormone-refractory tumour has low pAR^210^ nuclear expression (solid line) (*P*=0.003). (**B**) Kaplan–Meier plot demonstrates that those patients whose hormone-refractory tumour has high pAR^210^ nuclear expression (broken line) have shorter disease-specific survival than those patients whose hormone-refractory tumour has low pAR^210^ nuclear expression (solid line) (*P*=0.014).

When expression levels of each protein investigated in the hormone-refractory tumours were divided into high or low expression (levels above or below the median), only pAR^210^ was associated with quicker time to death from relapse (Figure 2A; *P*=0.003, HR=2.85 (95% CI: 1.38–5.87)) and quicker disease-specific survival (Figure 2B; *P*=0.0136, HR=2.33 (95% CI: 1.16–4.66). Median survival from time of relapse for those patients with tumours that expressed low levels of pAR^210^ was 3.42 (IQR: 2.82–4.02) years compared to 1.40 (IQR: 0.85–1.95) years for those who had tumours that expressed high levels of pAR^210^, and the median disease-specific survival for those patients with tumours that expressed low levels of pAR^210^ was 8.57 (IQR: 5.41–11.73) years compared to 5.82 (IQR: 3.18–8.46) years for those who had tumours that expressed high levels of pAR^210^. This represents a survival difference of almost 3 years for patients expressing high levels of pAR^210^ in their hormone-refractory tumour.

#### Are changes in protein expression in the transition from hormone-sensitive to -refractory disease associated with relapse or survival?

**Figure 3:**
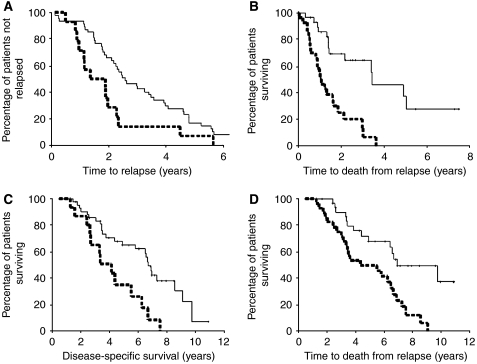
(**A**) Kaplan–Meier plot demonstrates that those patients whose tumours exhibit a rise in PI3K expression (broken line) relapse quicker than those patients whose tumours exhibit no change or a fall in PI3K expression (solid line). (**B**) Kaplan–Meier plot demonstrates that those patients whose tumours exhibit a rise in pAR^210^ expression (broken line) have shorter time to disease-specific death from time of biochemical relapse than those patients whose tumours exhibit no change or a fall in pAR^210^ expression (solid line). (**C**) Kaplan–Meier plot demonstrates that those patients whose tumours exhibit a rise in pAkt^473^ cytoplasmic expression (broken line) have shorter time to disease-specific death than those patients whose tumours exhibit no change or a fall in pAkt^473^ expression (solid line). (**D**) Kaplan–Meier plot demonstrates that those patients whose tumours exhibit a rise in pAR^210^ expression (broken line) have shorter time to disease-specific death than those patients whose tumours exhibit no change or a fall in pAR^210^ expression (solid line).

When expression levels in hormone-sensitive or -refractory tumours were used to investigate a link between activation of the PI3K pathway and development of hormone-refractory disease, only AR phosphorylated at the Akt consensus site was associated with survival. However due to the nature of the current cohort, we were also able to investigate if those patients whose tumours exhibit an increase or rise in expression of members of the pathway in the transition from hormone-sensitive to -refractory disease were more likely to relapse or die quicker. The cutoff histoscore selected to separate subgroups of patients is displayed in Table 1. Using this method, an increase in PI3K (Figure 3A; *P*=0.014, HR=2.11 (95% CI: 1.14–3.91)) was associated with quicker time to relapse. The median time to relapse for those patients whose tumours have a decrease or no change in PI3K expression was 2.57 (IQR: 1.74–3.40) years compared to 1.36 (IQR: 1.20–2.72) years for those patients whose tumours had an increase in PI3K expression.

A rise in pAR^210^ (Figure 3B; *P*<0.0001, HR=4.18 (95% CI: 1.99–8.74)) was associated with quicker time to death from relapse, the median survival from biochemical relapse for those patients whose tumours had a decrease or no change in pAR^210^ expression was 3.46 (IQR: 1.39–5.53) years compared to 1.25 (IQR: 0.83–1.67) years for those patients whose tumours had an increase in pAR^210^ expression.

A rise in pAkt^473^ (Figure 3C; *P*=0.0019, HR=2.89 (95% CI: 1.43–5.8)) and pAR^210^ (Figure 3D; *P*=0.0015, HR=2.86 (95% CI: 1.45–5.67)) were associated with shorter disease-specific survival. The median survival from diagnosis for those patients whose tumours had no change or decrease in pAkt^473^ was 6.68 (IQR: 6.22–7.14) years compare to 4.15 (IQR: 2.65–65) years for those patients whose tumours had an increase in pAkt^473^ expression, and the median survival from diagnosis for those patients whose tumours had a decrease or no change in expression of pAR^210^ was 6.95 (IQR: 4.07–9.83) years compared to 4.36 (IQR: 1.67–7.10) years for those patients whose tumours had an increase in pAR^210^ expression. Therefore, an increase in expression in the transition from hormone-sensitive to -refractory disease of phosphorylated members of this pathway was associated with a reduction in median survival of 2.5–3 years.

#### Correlations between active members of the pathway

**Figure 4:**
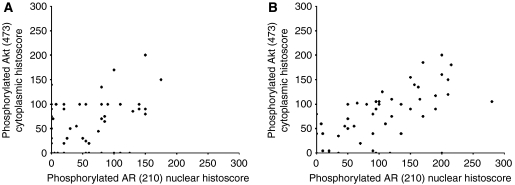
Scatter plots of pAkt^473^ histoscore compared to pAR^210^ histoscore, (**A**) is in the hormone-sensitive tissue and no significant correlation was observed (*P*=0.061, correlation coefficient 0.251); however, (**B**) is in the hormone-refractory tissue, where a significant correlation was observed (*P*<0.001 and correlation coefficient 0.711).

In hormone-sensitive tumours, expression levels of the phosphorylated proteins did not correlate; no correlation was made between Akt activation and AR phosphorylation (Figure 4A). However, in the hormone-refractory tumours pAkt^473^ correlated with pAR^210^ (*r*_s_=0.711, *P*<0.001) (Figure 4B) and pmTOR^2448^ (*r*_s_=0.489, *P*=0.003). Table 2 shows the individual histoscores for pAkt and PI3K in patients with and without pAR^210^ increases; no changes in median expression levels was observed between the groups.

## DISCUSSION

Cell line studies demonstrate that the PI3K cascade may influence the development of HRPC, suggesting this pathway may provide a novel therapeutic target for prostate cancer. However, to translate this into the clinic, we are required to provide evidence that this pathway is upregulated in the development of clinical HRPC and also identify what proteins would make the best targets and most effectively identify patients suitable for therapy.

It is well established in the literature that there is a link between Akt activation and development of HRPC. Cell line work demonstrates that Akt activity increases during androgen ablation to stimulate cell growth and survival when androgen reliance is weaker, and therefore promote development of HRPC (Gao *et al*, 2003; Ghosh *et al*, 2003; Lin *et al*, 2003). Work using human prostate tissue confirms that pAkt^473^ is expressed in PIN and invasive prostate cancer, and staining intensity positively correlated with PSA levels and Gleason grades (Ghosh *et al*, 2003; Altomare and Testa, 2005; Majumder and Sellers, 2005). In addition, a large study of 640 radical prostatectomy specimens demonstrated that high levels of pAkt was predictive of biochemical recurrence (Ayala *et al*, 2003). Although in our current cohort we did not observe a significant association with pAkt^473^ and biochemical recurrence (pAkt^473^; *P*=0.151, results not shown), the Kaplan–Meier curves did separate and the difference in the median time to biochemical recurrence in the two groups was 1.8 year compared to 2.4 years, suggesting that significance might have been meet if a larger cohort had been used. As the previous study was conducted on a cohort of 640 patient samples and our cohort contained only 68 patients, we performed a power calculation to assess what cohort size would have been required. This calculation suggested that a cohort of 200 patients would have been sufficient to reach significance. It is therefore not surprising that a significant result was achieved on a cohort of 640 samples. However the design of our study was to mirror the cell line experiments and investigate if changes in protein expression of members of the PI3K/Akt cascade were involved in the transition from hormone-sensitive to -refractory disease; therefore, we felt that it was not necessary to increase that cohort size. The strength of this study was the use of the paired samples, by doing so we observed that an increase in expression of multiple members of the pathway was linked to time to recurrence and disease-specific survival. In addition, if only primary tumours had been used, we would not have observed that pAR^210^ was linked to survival, as marked expression was only observe in hormone-refractory tumours. In the current study, we observe that an increase in pAkt^473^ expression, fully activated Akt and an increase in pAR^210^ was associated with decreased survival. Providing further evidence that activation of the PI3K/Akt cascade is associated with development of HRPC.

**Figure 5:**
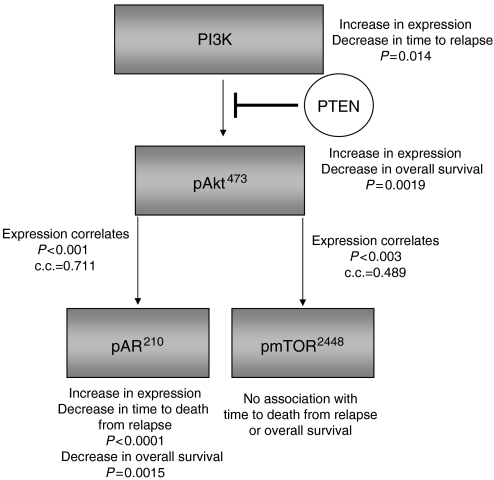
A simplified cartoon of the PI3K/Akt pathway; the *P*-values and correlation coefficients (*r*_s_) represent those found when protein expression/activation were correlated in HRPC tissue specimens.

Cell line data suggest that phosphorylation of AR by Akt at serine 210 results in an increase in AR translational activity when androgen levels are low, and, in addition, activated Akt, over a longer period, has been demonstrated to upregulated AR expression in LNCaP cells (Manin *et al*, 2002; Yang *et al*, 2005). The impact of both these actions of Akt on the AR will serve to increase the sensitivity of the AR to androgens, enabling transcription of AR-regulated genes even when androgens levels are very low, similar to those experienced during chemical or surgical castration. This therefore provides a mechanism for the development of HRPC. In the current cohort, HRPCs express significantly higher levels of pAR^210^ compared to hormone-sensitive tumours; the median histoscore of refractory tumours is 103 histoscore units compared to 35 histoscore units for sensitive tumours, *P*=<0.0001. Approximately 42% of patients have an increase in pAR^210^ expression, and these patients have a significantly shorter survival period than those with no increase in expression. In addition, expression levels of pAR^210^ and AR strongly correlate with pAkt^473^ expression levels only in the refractory tumours, suggesting that it is only when androgen levels are low that this pathway is activated. These results provide additional evidence that the PI3K/Akt pathway is upregulated during development of HRPC, resulting in phosphorylation of the AR and sensitisation to circulating adrenal androgens. Cell line studies demonstrate that this occurs *in vitro*; however, the current data demonstrate for the first time that this may be one possible mechanism allowing development of HRPC in the clinical setting (Figure 5).

The protein mTOR is also downstream of Akt and was also investigated in this study as it has previously been to HRPC (Bubley *et al*, 2003). Akt can phosphorylate mTOR directly at threonine 2446 and serine 2448, but can also activate mTOR indirectly via phosphorylation of TSC2 (Majumder and Sellers, 2005). In the current cohort, pAkt^473^ expression correlates with pmTOR^2448^ expression; however, expression levels or changes in expression levels of pmTOR^2448^ do not correlate with any clinical parameters in our cohort. This suggests that mTOR may not be involved in the development of HRPC. This was surprising as stimulation of mTOR ultimately results in increase protein synthesis and enhances translation of proteins involved in growth control via turning off 4EBP and activating S6Kinase. Although Akt has been demonstrated to phosphorylate mTOR directly, the role of these phosphorylation sites remains unclear. Phosphorylation of mTOR by Akt at serine 2448 might not correlate with mTOR activation, therefore, pmTOR^2448^ may not necessary be involved with the development of HRPC, and a more appropriate marker of mTOR activation could be S6Kinase (Ruggero and Sonenberg, 2005).

In summary, they current study demonstrates a role for the PI3K/Akt/AR pathway in the development of HRPC; however, now that drugs are being developed to target specific components of pathways, it is necessary to identify the proteins in the individual pathways that would make the most appropriate targets. Results from the current study suggest that a larger portion of tumours would respond to drugs targeting Akt activation in contrast to PI3K or PTEN. Methods currently being explored for inhibition of Akt activation include Akt antibodies, similar to humanised trastuzamab (Herceptin), ATP-competitive inhibitors and PDK1 inhibitors (Cheng *et al*, 2005). In addition to Akt being the appropriate target for therapy, Akt in combination with pAR^210^ might be the appropriate marker for identifying patients most likely to respond to drugs that target Akt activation. Phosphorylation of Akt and AR are clearly linked with decreased survival, those patients who have an increase in pAtk^473^ and/or pAR^210^ have median overall survival period of 3.98 years compared to 6.68 years, this is a survival difference of almost 3 years, and approximately 50% of the patients in our study have an increase in pAkt^473^ and/or pAR^210^ expression, suggesting that the majority of patients likely to respond to Akt inhibition will be identified by these markers. This study emphasises the need for a rational approach for new drug design, and the progress already made in breast cancer demonstrates the effectiveness of such an approach.

## Figures and Tables

**Table 1:** Histoscore variation and comparison of staining intensity for hormone-sensitive and hormone-refractory tumours

	**HSPC (IQR)**	**HRPC (IQR)**	***P*-value**	**ICCC**	**Histoscore units**	**Fallers**	**Risers**
PI3Kc	100 (58–140)	100 (79–134)	0.875	0.85	60	23	23
Akt1m	0 (0–11)	0 (0–90)	0.798	0.88	30	14	13
Akt1c	75 (20–100)	70 (0–90)	0.488	0.82	55	20	13
Akt1n	0 (0–18)	0 (0–0)	0.110	0.95	21	19	9
Akt2	125 (100–185)	120 (98–165)	0.551	0.81	61	22	13
Akt3	50 (0–100)	60 (0–95)	0.619	0.84	48	20	30
pAkt^473^m	40 (0–90)	33 (0–90)	0.988	0.90	58	22	23
pAkt^473^c	88 (54–110)	80 (40–105)	0.671	0.89	49	25	23
pAkt^473^n	0 (0–25)	0 (0–35)	0.465	0.93	40	15	18
pAR^210^n	35 (0–85)	103 (50–169)	**<0.0001**	0.93	52	8	42
mTORm	0 (0–20)	0 (0–10)	0.134	0.92	47	10	3
mTORc	43 (15–87)	40 (10–62)	0.123	0.83	31	35	23
pmTOR^2448^m	0 (0–22)	0 (0–15)	0.330	0.95	33	14	8
pmTOR^2448^c	61 (20–100)	40 (8–70)	0.044	0.91	45	33	23
pmTOR^2448^n	0 (0–10)	0 (0–0)	0.575	0.90	19	3	6

Table 1 shows the median histoscore and interquartile range (IQR) for hormone-sensitive tumours (HSPCs) and hormone-refractory tumours (HRPCs) and the *P*-values of HSPC histoscores compared to HRPC histoscores using a Wilcoxon signed-rank test. The median histoscore was the cutoff histoscore used to select for the separate subgroups when defining high and low expressers. The interclass correlation coefficient (ICCC), which measures consistence between observers for each protein, is consistently higher than 0.7, which is classed as excellent. The mean difference in observer scores plus 2 s.d. is also shown as the number of histoscore units, that is, the cutoff in histoscore units used to select for the separate subgroups, which is defined as a change in protein expression. The percentage of tumours that were defined as having a fall or rise in protein expression (calculated using the number of histoscore units, which is defined as a change in expression) are also shown. ‘m’, ‘c’ and ‘n’ relates to protein location, m=membrane, c=cytoplasm and n=nucleus. ‘P’ before a protein indicates that the antibody detects phosphorylated protein, and the number following the protein represents the site of phosphorylation. Bold value represents statistical significance.

**Table 2:** Histoscores of pAkt and PI3K and comparison of staining intensity between patients with or without an increase in pAR^210^ expression

**Group 1**				**Group 2**			
**HS**		**HR**		**HS**		**HR**	
**PAkt**	**PI3K**	**pAkt**	**PI3K**	**pAkt**	**PI3K**	**pAkt**	**PI3K**
0	80	0	70	0	30	110	95
0	100	105	90	70	110	NA	180
90	160	0	60	50	80	NA	105
0	260	0	100	55	50	100	40
NA	NA	NA	NA	105	105	NA	160
130	140	55	60	90	105	180	80
90	20	125	90	0	130	40	90
NA	NA	120	160	25	30	140	100
50	15	NA	100	75	190	2.5	280
130	70	105	0	110	200	55	35
50	53.75	60	100	85	25	150	10
110	200	0	132.5	100	40	5	180
NA	NA	NA	NA	100	80	117.5	130
135	130	40	140	80	25	90	105
20	100	102.5	170	95	140	0	62.5
130	105	50	120	97.5	100	75	90
NA	100	NA	30	72.5	115	105	100
100	0	5	50	0	0	110	70
110	180	80	195	100	0	NA	NA
100	100	20	200	75	110	120	115
100	195	100	165	65	80	185	100
162.5	190	90	100	0	15	90	100
180	210	100	160	NA	NA	NA	NA
NA	90	NA	120	137.5	170	35	105
165	0	40	100	0	90	75	75
60	40	5	25	NA	200	NA	137.5
85	190	80	0	160	200	135	150
25	115	100	100	125	170	0	190
90	100	155	80	220	200	0	150
35	100	70	25	120	120	NA	160
70	120	40	100	72.5	100	100	80
85	NA	60	NA	75	40	130	0
NA	200	NA	100	120	80	150	0
90	100	60	100	NA	NA	NA	NA

NA=not available. Table 2 shows the histoscore for cytoplasmic phosphorylated Akt and PI3K in hormone-sensitive tumours (HS) and hormone-refractory tumours (HR). Group 1 represents those patients with no increase in phosphorylated AR expression, and group 2 represents those patients with an increase in phosphorylated AR expression in the transition from hormone-sensitive to hormone-refractory disease.
